# In Situ Construction of Multifunctional Organosilicon/Organic Hybrid Resin Coatings on Wood Surfaces Based on the PMHS Si–H Click Reaction

**DOI:** 10.1021/acsomega.6c05204

**Published:** 2026-07-20

**Authors:** Zhixuan Ye, Xingshu Chen, Linxin Zhang, Jun Wang, Hanxian Chen, Xinxiang Zhang, Weiwei Zhao, Zhanhui Yuan

**Affiliations:** † College of Materials Engineering, 12449 Fujian Agriculture and Forestry University, Fuzhou 350108, China; ‡ Birmingham Centre for Energy Storage (BCES) & School of Chemical Engineering, 1724 University of Birmingham, Edgbaston B15 2TT, U.K.; § Fujian Key Laboratory of Functional Marine Sensing Materials, College of Materials and Chemical Engineering, 26465 Minjiang University, Fuzhou 350108, China

## Abstract

The abundant hydrophilic hydroxyl groups and porous structure make wood highly susceptible to moisture absorption from the environment, leading to issues such as deformation and decay. This work aims to in situ construct a multifunctional organosilicon/organic hybrid resin coating on the wood surface derived from the click reaction of polymethylhydrosiloxane (PMHS), using PMHS, divinylbenzene (DVB), and the Karstedt catalyst. At room temperature, through the Si–H addition click reaction between PMHS and DVB, a cross-linked and rigid hybrid resin coating with a hardness of 94.00 HA is fabricated on the wood surface. Simultaneously, a dehydrogenation click reaction takes place between the Si–H groups of PMHS and the –OH groups on the wood surface, anchoring the hybrid resin coating to the wood via covalent bonds. These endow the hybrid resin coating with excellent mechanical stability. The low surface energy of organosilicon resin in the hybrid coating affords the modified wood with good waterproofness by decreasing the 24 h water absorption rate from 78.6% to 13.9%. Most importantly, the coating remains hydrophobic even after being immersed in an alkaline solution (pH = 13) for 24 h. SEM images of the coating after alkaline corrosion indicate that while the Si–O–Si skeleton of the organosilicon component is partially dissolved due to its susceptibility to alkali, the hydrophobic organic resin skeleton remains intact, imparting good alkali resistance to the hybrid resin coating. In summary, the organosilicon/organic hybrid coating constructed via the click reaction of PMHS on the wood surface effectively addresses the issue of poor alkali resistance commonly associated with traditional organosilicon coatings.

## Graphical abstract

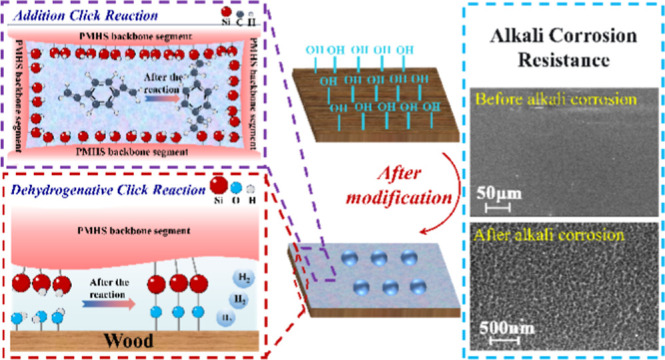

## 1 Introduction

As a natural renewable material, wood is widely used in architectural decoration, furniture manufacturing, and timber construction due to its high specific strength, lightweight, and robustness.^1–5^ However, wood is primarily composed of cellulose, hemicellulose, and lignin. Cellulose and hemicellulose contain abundant polar hydroxyl groups, which readily form hydrogen bonds with water molecules, resulting in strong hydrophilicity. Additionally, the wood structure features numerous pores and capillary channels, allowing moisture to penetrate rapidly via capillary action. This combination of a polar porous structure and strongly hydrophilic hydroxyl groups makes wood highly susceptible to moisture absorption and swelling, leading to deformation, cracking, decay, and mold growth, which severely limits its service life and application scope.^6–9^ Therefore, effective waterproofing and protective treatment of wood are of great significance.^10,11^

Current wood protection methods can be mainly categorized into thermal modification,^12–14^ chemical modification,^15–17^ and wood preservative treatment.^18^ Among these wood modification methods, wood preservative treatment can effectively enhance the resistance of wood to biodegradation through chemical means, but it cannot effectively improve the inherent physical deficiencies of wood. In addition, most preservatives have problems such as high toxicity, potential environmental residues, or limited applicable conditions, which significantly limit their sustainability and safety.^18^ In contrast, wood modification represents a direct and efficient protection strategy, substantially improving moisture resistance and decay resistance. Among the modification approaches, thermal modification^12–14^ and chemical modification techniques such as furfurylation^15^ and acetylation^16^ are widely employed for wood protection. However, the backbone of modifiers or protective agents used in these methods is predominantly based on C–C or C–O. The bond energies of C–C and C–O bonds are approximately 332 kJ·mol^–1^ and 326 kJ·mol^–1^, respectively, and therefore, these bonds are prone to cleavage under ultraviolet (UV) irradiation, leading to a short service life for this modified wood.^19^

Silicone materials exhibit excellent weather resistance, resistance to high and low temperatures, and stain resistance due to the high bond energy of the Si–O bonds (454 kJ·mol^–1^) in their backbone, making them widely used for constructing long-lasting hydrophobic coatings on various wood surfaces.^20–22^ Commonly employed silicone materials include silane coupling agents,^23,24^ silicone rubbers,^25^ and silicone resins.^26,27^ Silane coupling agents can form a hydrophobic siloxane network within the wood cell wall via hydrolysis and condensation, thereby improving hydrophobicity, dimensional stability, and decay resistance.^23,24^ Silicone rubbers are frequently used to prepare flexible coatings or as impregnating agents; their good moisture resistance and elasticity can accommodate wood swelling and shrinkage, achieving durable waterproofing.^25^ However, the mechanical stability of the coating formed by the silane coupling agent and silicone rubber is still insufficient, making it unsuitable for applications requiring wear resistance. Silicone resins can form hard, transparent, and weather-resistant hydrophobic protective films on wood surfaces, effectively mitigating the impact of environmental moisture and ultraviolet radiation on wood.^26,27^ Clearly, silicone coatings offer superior weather resistance compared to traditional coatings, making them suitable for outdoor wood protection. However, the Si–O–Si backbone of silicone is readily dissolved by NaOH solutions.^28^ Consequently, silicone coatings exhibit poor alkali resistance, which is a critical issue that must be addressed for the development of multifunctional silicone coatings.

In this work, an organosilicon/organic hybrid resin coating is constructed in situ on wood via click reactions among PMHS, DVB, and wood. With the Karstedt catalyst, the addition click reaction between the Si–H bonds of PMHS and the vinyl groups of DVB enables efficient curing of the hybrid resin. Concurrently, the dehydrogenation click reaction between the Si–H bonds of PMHS and the –OH groups on the wood surface establishes covalent bonding between the hybrid coating and the wood substrate. These two click reactions ensure the structural integrity and interfacial stability of the hybrid coating, effectively prolonging its service life. As noted above, silicone coatings suffer from poor alkali resistance. In an alkaline environment, although the organosilicon Si–O–Si backbone is dissolved by the alkali (i.e., the silicone resin is degraded), the densely cross-linked hydrophobic organic resin framework remains structurally intact, continuing to resist the erosion of the wood by the alkaline aqueous solution. This imparts favorable alkali resistance to the hybrid coatings. The organosilicon/organic hybrid coating constructed via the reactive hybridization of PMHS and DVB on the wood surface combines the UV resistance and stain resistance of silicone with the alkali resistance and wear resistance of organic resins, overcoming the challenges of coatings from pure organic resins or organosilicon resins.

## 2 Experimental Section

### 2.1 Materials

Unsanded Chinese fir blocks (local construction materials market, Fujian, China) measuring 25 mm­(r) × 25 mm­(t) × 10 mm­(l)) were oven-dried (100 °C, 24 h) before surface modification. PMHS with an active hydrogen (Si–H) content of 1.5% was supplied by China Bluestar Chengrand Co., Ltd. DVB was purchased from Shanghai Aladdin Biochemical Technology Co., Ltd. A Karstedt catalyst (platinum-1,3-divinyl-1,1,3,3-tetramethyldisiloxane) with a platinum concentration of 7500 ppm was obtained from China Bluestar Chengrand Co., Ltd. Inorganic pigments (yellow, red, green, blue) with a particle size of 0.4 μm were provided by Shanghai Yipin Pigment Co., Ltd. All chemicals were used as received without further purification.

### 2.2 Preparation of Hybrid Resin

PMHS and DVB with different weight ratios and the Karstedt catalyst (Table 1) were sequentially added into a beaker and stirred at room temperature for 1 min, which was then poured into a mold and cured at room temperature to obtain the organosilicon/organic hybrid resin.

**Table 1:** Formulations for Preparation of Organosilicon/Organic Hybrid Resin

weight ratio of PMHS to DVB	PMHS/g	DVB/g	Karstedt catalyst/g
1:2	4	8	0.1
1:1	6	6	0.1
2:1	8	4	0.1
3:1	9	3	0.1
4:1	9.6	2.4	0.1
5:1	10	2	0.1
6:1	10.2	1.7	0.1

### 2.3 Preparation of the Hybrid Resin Coating

According to Table 1, PMHS and DVB with different weight ratios were mixed together with the Karstedt catalyst, which were then sprayed onto the wood surface with an airbrush, respectively. The application rate of hybrid coating on the wood surface was 100 g·m^–2^ unless otherwise specified. The spraying amount of the hybrid coating was 100 unless otherwise specified. After curing at different temperatures with varying curing times, the modified wood with the organosilicon/organic hybrid resin coating was obtained.

### 2.4 Characterization

The hardness of the modified wood was measured using a Shore hardness tester (Model LX-A, Nanjing Suce Measurement Instrument Co., Ltd., Nanjing, China). The surface morphology of the wood samples, both before and after modification, was examined by field emission scanning electron microscopy (FESEM, Gemini SEM 500, Carl Zeiss (Shanghai) Co., Ltd., Shanghai, China) at an acceleration voltage of 3 kV. Prior to observation, a thin gold (Au) layer was sputtered onto the sample surfaces for 100 s to enhance conductivity. Changes in the surface chemical composition of the wood were analyzed using Fourier transform infrared spectroscopy (FTIR, Bruker Tensor 27) and X-ray photoelectron spectroscopy (XPS, Thermo Scientific Escalab 250Xi). The FTIR spectra were recorded over the range of 4000–400 cm^–1^ at a resolution of 4 cm^–1^, with 32 scans. The static water contact angle (WCA) on the modified wood was measured using a contact angle meter (HARKE-SPCA-1, Beijing Harke Testing Equipment Co., Ltd., Beijing, China) at room temperature with a distilled-water drop volume of 5 μL. The WCA was recorded 5 s after the drop was placed on the surface. To ensure reliability, the WCA was measured at five different points on each sample, and the average value was reported.

To evaluate the water absorption and dimensional stability of the modified wood, 24 h water uptake was measured. The initial mass of each specimen was recorded, and the mass was measured again after 24 h of submersion in water. The water absorption rate was calculated according to eq 1

1 $$w_{m}=\left(m_{1}-m_{0}\right)/m_{0}\times 100\%$$

*w*_m_: water absorption; *m*_0_: the initial mass of the wood specimen before immersion; *m*_1_: the mass of the wood specimen after immersion. Furthermore, the mechanical stability of the modified wood was examined through a sandpaper abrasion test.^29^ Specifically, under a constant load provided by a 200 g weight, the surface coating was subjected to friction with 1500-grit sandpaper. The WCA was measured after every 20 cm of abrasion distance. For the corrosion resistance test, the modified wood was immersed in solutions with pH values of 1, 3, 5, 9, 11, and 13 for durations of 0, 3, 6, 12, and 24 h. After immersion, the samples were rinsed with deionized water, dried in an oven at 100 °C, and their WCAs were measured. To assess the UV aging resistance, the specimens were exposed to 36 W ultraviolet light with a wavelength of 356 nm at a vertical distance of 20 cm. The WCA was measured at 30 min intervals. The UV irradiation was paused when the WCA dropped below 90° and was resumed after the WCA recovered above 90°, with this cycle being repeated. Finally, the optical transmittance of the coating was measured using a UV–visible spectrophotometer (UV-6100, Shanghai Mapada Instruments Co., Ltd., Shanghai, China).

## 3 Results and Discussion

### 3.1 Fabrication Process of the Hybrid Resin Coating

Figure 1a–d illustrates the preparation process and mechanism of the organosilicon/organic hybrid resin and the resultant coating on the wood surface. As shown in Figure 1a, DVB and PMHS were mixed in a different weight ratio, followed by the addition of the Karstedt catalyst with thorough stirring to obtain the hybrid resin solution. The solution was cured under ambient conditions to form the organosilicon/organic hybrid resin (Figure 1c). The coating solution was also applied onto the wood surface via spraying, and after curing, modified wood with an organosilicon/organic hybrid resin coating was obtained. The underlying mechanism of the hybrid coating can be intuitively elucidated at the microscopic level. As shown in Figure 1b, with the Karstedt catalyst, the Si–H bonds of PMHS undergo a dehydrogenation click reaction with the –OH groups on the wood surface to form covalent bonds,^26^ enabling the hybrid coating to be tightly bonded to the wood substrate via chemical bonds, thereby significantly enhancing the mechanical stability of the hybrid coating. Simultaneously, the Si–H bonds of PMHS undergo an addition click reaction with the vinyl groups of DVB (Figure 1d), constructing a three-dimensional hybrid network of organosilicon/organic hybrid resin on the wood surface. The hydrophobic –CH_3_ groups carried by PMHS are uniformly distributed within this network, endowing the hybrid coating with low surface energy and hence high hydrophobicity and antifouling. The FTIR spectra further corroborated this process. As shown in Figure 1e, compared with untreated wood, the intensity of the absorption band attributed to –OH groups at 3421 cm^–1^ is significantly weakened,^30,31^ confirming that the Si–H bonds of PMHS underwent a dehydrogenation click reaction with the –OH groups on the wood surface.^26^ Meanwhile, new absorption bands appear at 2968 cm^–1^, 2164 cm^–1^, 767 cm^–1^, and 844 cm^–1^. The bands at 2968 cm^–1^ and 2164 cm^–1^ correspond to the stretching vibrations of Si–CH_3_ ^26^ and Si–H of PMHS, respectively. The band at 767 cm^–1^ is attributed to the bending mode of Si–O–Si,^27,32^ and the band at 844 cm^–1^ arises from the stretching vibration of the benzene ring of DVB. These results indicate that PMHS and DVB were successfully introduced onto the wood surface, and an organosilicon/organic hybrid resin coating was constructed in situ on the wood surface.

**Figure 1:**
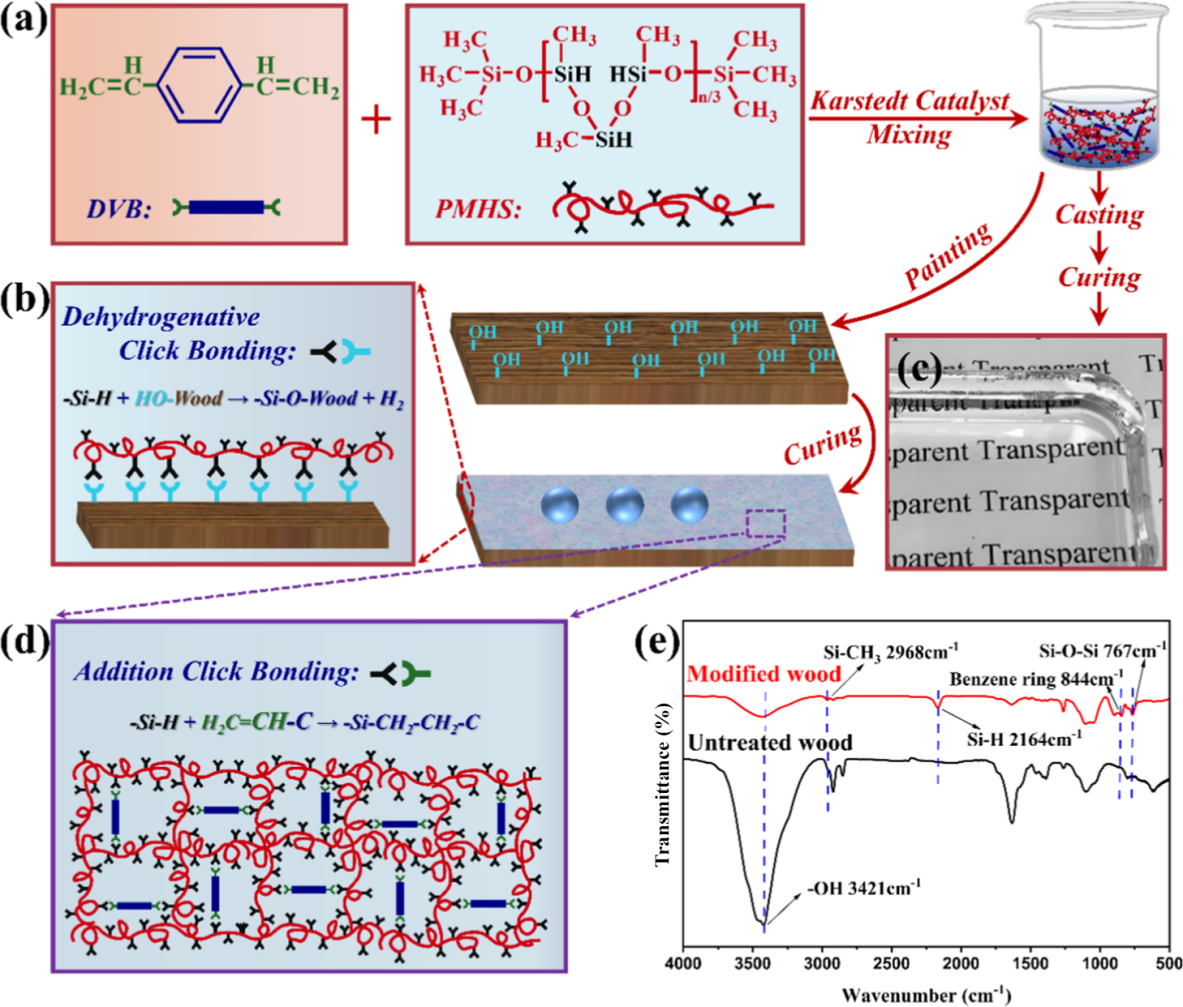
(a) Fabrication process diagram of the organosilicon/organic hybrid resin coating. (b) Schematic diagram of the dehydrogenation click reaction between the coating and the wood substrate. (c) Cured organosilicon/organic hybrid resin. (d) Schematic diagram of the addition click reaction between PMHS and DVB. (e) FTIR spectra of wood before and after modification.

### 3.2 Hardness of the Organosilicon/Organic Hybrid Resin

The hardness of the organosilicon/organic hybrid resin significantly affects the abrasion resistance of the hybrid coating on the wood surface, and its hardness is related to the cross-linking density of the hybrid resin. Figure 2a–d shows the effects of the PMHS/DVB mass ratio and curing time on the hardness of the hybrid resin. As shown in Figure 2a, when the PMHS/DVB mass ratios were 1:2 and 1:1, the hybrid resin remained in a gel state and no valid hardness could be measured. Thus, the hardness value was recorded as 0. From Figure 2a-d, it can be observed that at different curing times, the hardness of the hybrid resin initially increased and then decreased with an increasing PMHS/DVB mass ratio. When the PMHS/DVB mass ratio was 3:1, the hybrid coating had the highest hardness value because it had the highest cross-linking degree. The cross-linking degree of the hybrid resin is highly related to the addition click reaction between the Si–H bonds of PMHS and the vinyl groups of DVB and hence the mass ratio of PMHS to DVB. A suitable mass ratio of PMHS to DVB will give the highest cross-linking density to the hybrid coating. PMHS is the cross-linker for the fabrication of the hybrid resin coating. At a low mass ratio of PMHS to DVB, the increasing addition of PMHS gradually increases the cross-linking density. However, excessive PMHS leads to insufficient cross-linking during the hybridization process of the hybrid coating due to the inadequate dosage of DVB. Therefore, the mass ratio of PMHS to DVB is selected to be 3:1 in the fabrication of the hybrid coating on wood.

**Figure 2:**
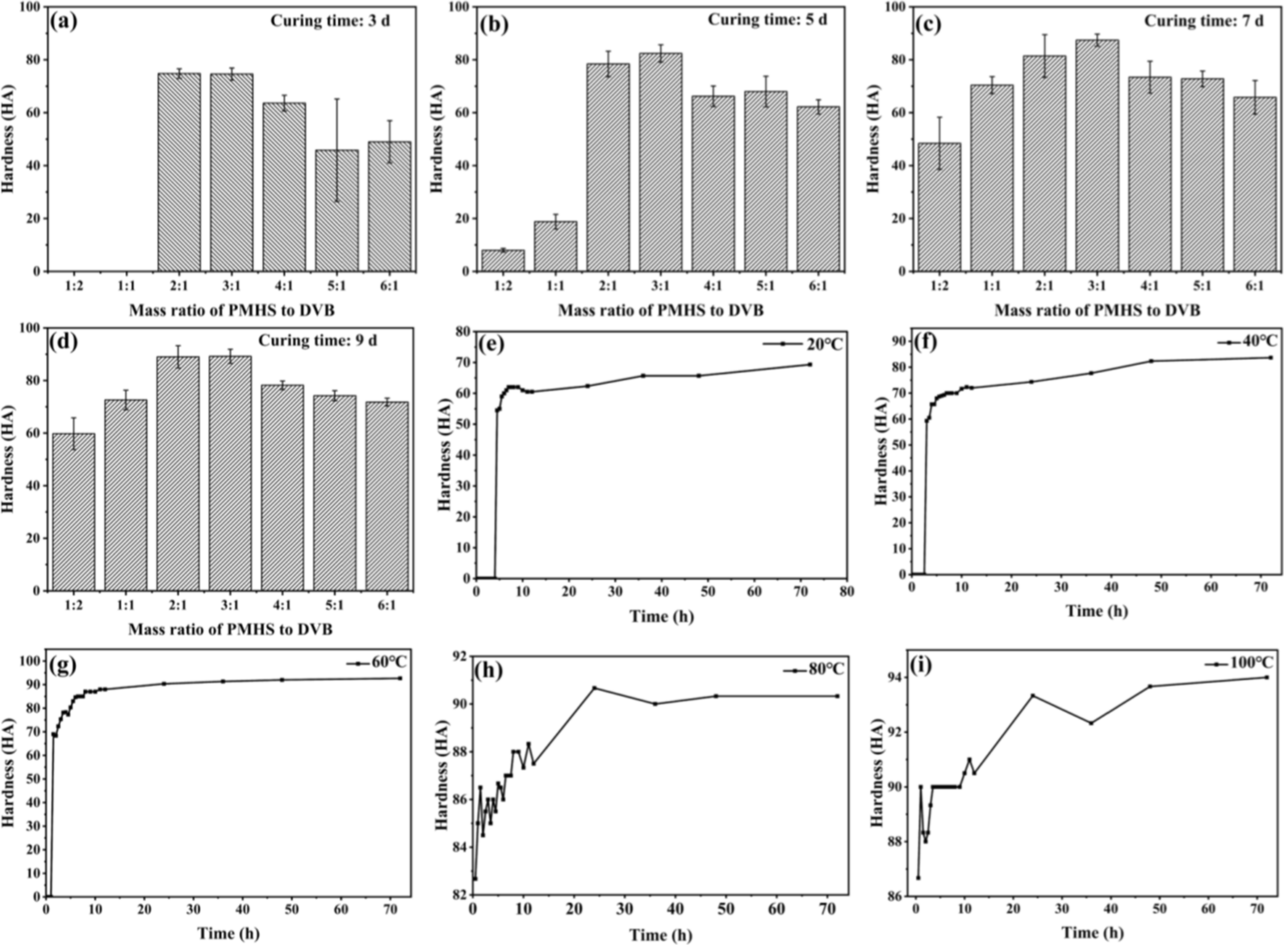
Effect of the PMHS/DVB mass ratio and curing time at room temperature on the hardness of the organosilicon/organic hybrid resin (curing time: (a) 3 days, (b) 5 days, (c) 7 days, and (d) 9 days). The hardness of the organosilicon/organic hybrid resin as a function of curing time at different curing temperatures ((e) 20 °C, (f) 40 °C, (g) 60 °C, (h) 80 °C, and (i) 100 °C).

In addition, the hardness of the hybrid resin with different mass ratios increased with a prolonged curing time, exhibiting a self-reinforcing effect. As shown in Figure 2a–d, when the PMHS/DVB mass ratio was 3:1, the hardness of the hybrid resin increased from 74.6 HA to 89.2 HA as the curing time extended from 3 days to 9 days. This is because in the initial stage, only a portion of the Si–H bonds in PMHS reacted with the vinyl groups of DVB under the catalysis of the Karstedt catalyst, while a considerable number of Si–H bonds and vinyl groups remained unreacted due to steric hindrance, resulting in a relatively low cross-linking density. As the curing time increased, the flexible silicone chains underwent internal rotation, allowing the unreacted Si–H and vinyl groups to continue cross-linking, thereby further increasing the cross-linking density and endowing the hybrid resin with a self-reinforcing effect.

Curing temperature also affects the cross-linking density of the hybrid resin. Figure 2e–i illustrates the effects of curing temperature and curing time on the hardness of the hybrid resin. As shown in Figure 2e, when the curing time was between 0 and 4 h, the hybrid resin remained in a gel state, and the hardness could not be measured. Thus, the hardness value was recorded as 0. When the curing time ranged from 4.5 to 72 h, the hardness of the hybrid resin increased from 54.50 HA to 69.33 HA, further demonstrating the influence of curing time on hardness. Moreover, as the curing temperature increased, the hardness of the hybrid resin exhibited a gradual upward trend. As shown in Figure 2e–i, when cured at 20 °C for 72 h, the hardness of the hybrid resin was 69.33 HA. After increasing the curing temperature to 100 °C while maintaining the same curing time, the hardness of the hybrid resin increased to 94.00 HA. It is evident that both increasing the curing temperature and prolonging the curing time can effectively enhance the hardness of the hybrid resin, and the improvement effects are comparable, which aligns with the time–temperature superposition principle. Given that this hybrid coating is predominantly applied under ambient conditions, it can be inferred that its abrasion resistance will gradually improve with extended service time.

### 3.3 Comprehensive Performance of the Hybrid Resin Coating

Mechanical stability is very important for the protective coating fabricated on wood surfaces.^33^ The abrasion resistance of the hybrid coating critically affects its service life. Figure 3a shows the effect of curing time on the abrasion length of the hybrid coating (the abrasion length is defined as the distance over which the water contact angle of the hybrid resin coating gradually decreases from its maximum value to below 90° under sandpaper friction). As shown in Figure 3a, the abrasion length of the coating gradually increased with prolonged curing time. When the curing time was extended from 3 days to 30 days, the abrasion length increased from 1175 to 4300 cm. This is attributed to the fact that increasing the curing time significantly enhances the cross-linking degree of the hybrid resin, resulting in a substantial increase in coating hardness and consequently improved abrasion resistance. Figure 3b compares the abrasion resistance of different coatings. The total abrasion lengths of the silicone rubber coating and the silicone resin coating were 2250 and 2940 cm, respectively, while the total abrasion length of the prepared organosilicon/organic hybrid resin coating reached 4300 cm, demonstrating that the hybrid coating exhibits superior abrasion resistance.

**Figure 3:**
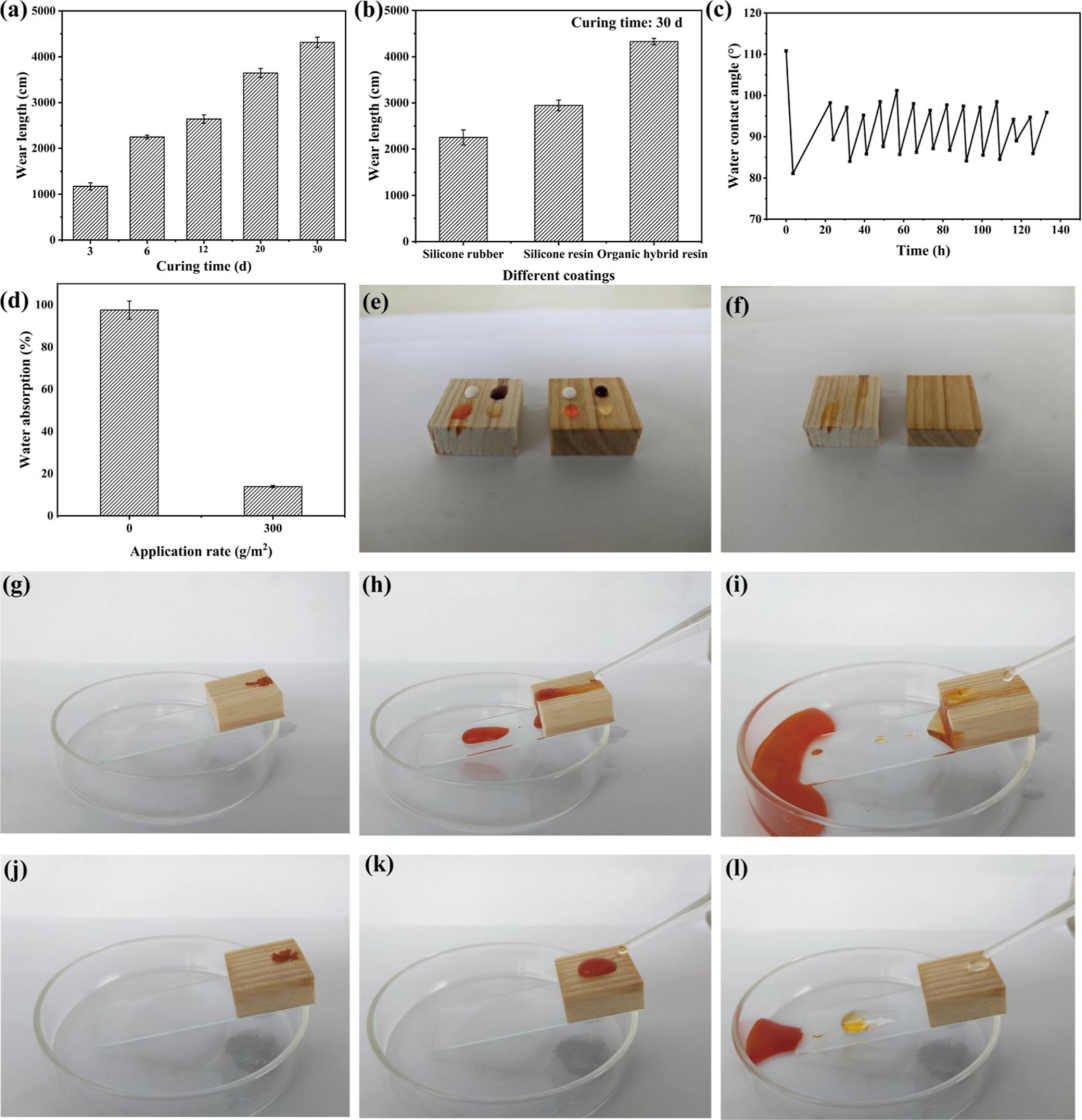
(a) Effect of curing time on the abrasion resistance of the hybrid coating (mass ratio of PMHS to DVB: 3:1). (b) Abrasion resistance of coatings from silicone rubber (prepared according to ref 25), silicone resin (prepared according to ref 26), and organic hybrid resin (mass ratio of PMHS to DVB: 3:1). (c) Effect of UV irradiation time on the hydrophobicity of the coating (mass ratio of PMHS to DVB: 3:1). (d) 24 h water absorption rates of wood coated with the hybrid coating at different application rates. (e–f) Stain resistance of wood before and after modification. Dye rinsing process of (g–i) pristine wood and (j–l) wood with the hybrid coating (mass ratio of PMHS to DVB: 3:1).

To further evaluate the weather resistance of the organosilicon/organic hybrid resin coating, UV aging resistance tests were conducted. Figure 3c shows the effect of UV irradiation time on the hydrophobicity of the hybrid coating. After 3.5 h of UV irradiation, the water contact angle of the modified wood decreased to 81.1°, exhibiting hydrophilicity. This may be attributed to the degradation of the organic framework within the hybrid coating under UV irradiation, generating hydrophilic hydroxyl groups, which consequently led to the loss of hydrophobicity.^34^ However, upon cessation of UV irradiation, the hydrophobicity of the modified wood gradually recovered. This may be ascribed to the flexibility of the polymer molecular chains.^35^ With extended storage time at room temperature, the polymer chains undergo rearrangement via internal rotation, allowing the hydrophobic Si–CH_3_ groups to migrate back to the coating–air interface, thereby restoring the hydrophobicity of the modified wood. The UV aging resistance test results indicate that the organosilicon/organic hybrid resin coating possesses favorable weather resistance and self-healing capability.

Wood is rich in hydrophilic hydroxyl groups, and its natural fibrous structure along with high porosity makes it highly susceptible to moisture absorption.^5^ Consequently, during storage and service, wood is prone to deformation, warping, cracking, and even mold growth due to repeated shrinkage and swelling. Therefore, improving the water resistance of wood is of great significance. Figure 3d shows the 24 h water absorption rates of wood before and after the modification. The data indicate that the water absorption rate of untreated wood after 24 h of immersion was as high as 78.6%, whereas that of the modified wood was only 13.9%, representing a significant reduction compared with untreated wood. This is attributed to the dehydrogenation click reaction between the abundant hydroxyl groups on the modified wood surface and the Si–H bonds in PMHS, which grafted a large number of hydrophobic methyl groups onto the wood surface, thereby endowing the modified wood with excellent water resistance.

Figure 3e–f illustrates the stain resistance of the wood surface with and without hybrid resin coating, in which water, milk, cola, and methyl orange solution were dripped onto their surface as pollutants. As shown in Figure 3e, on the pristine wood, all four liquids rapidly spread and infiltrated, whereas on the wood with a hybrid resin coating, all droplets maintained a regular spherical morphology due to its excellent hydrophobicity. In Figure 3f, the liquids are removed by absorbing them with tissue. Obvious stains remained on the surface of the untreated wood, while no traces were left on the modified wood surface, indicating the excellent antifouling property of the hybrid resin coating on wood.

Figure 3g–l shows the process of dye solution on the wood surface before and after modification being rinsed with water. As shown in Figure 3g–i, the dye on the untreated wood surface gradually spread and penetrated during the rinsing process, ultimately leaving clear dye marks on the wood surface. In contrast, the dye on the modified wood surface was completely carried away by the water flow, leaving no staining or water residue on the wood surface (Figure 3j–l), demonstrating that the modified wood surface possesses excellent stain resistance.

### 3.4 Corrosion Resistance of the Hybrid Resin Coating

The corrosion resistance of a coating is a critical factor affecting its actual service life. Figure 4a–f shows the changes in water contact angles of the modified wood after immersion in aqueous solutions with different pH values. As shown in Figure 4a, after immersion in an acidic solution with pH = 1 for 24 h, the water contact angle of the modified wood decreased from 112.9° to 101.7°, remaining above 90°, indicating good acid resistance of the coating. Furthermore, after immersion in an alkaline solution with pH = 13 for 24 h, the surface water contact angle of the modified wood decreased from 114.1° to 100.1° (Figure 4b), still maintaining good hydrophobicity, demonstrating that the hybrid coating possesses excellent alkali resistance. This is attributed to the fact that, although the organosilicon framework in the hybrid coating is degraded in the alkaline solution, the organic resin framework remains intact and adheres to the wood surface, enabling the modified wood to maintain good hydrophobicity. Consequently, the hybrid coating exhibits good alkali corrosion resistance, which can be further corroborated by the SEM images.

**Figure 4:**
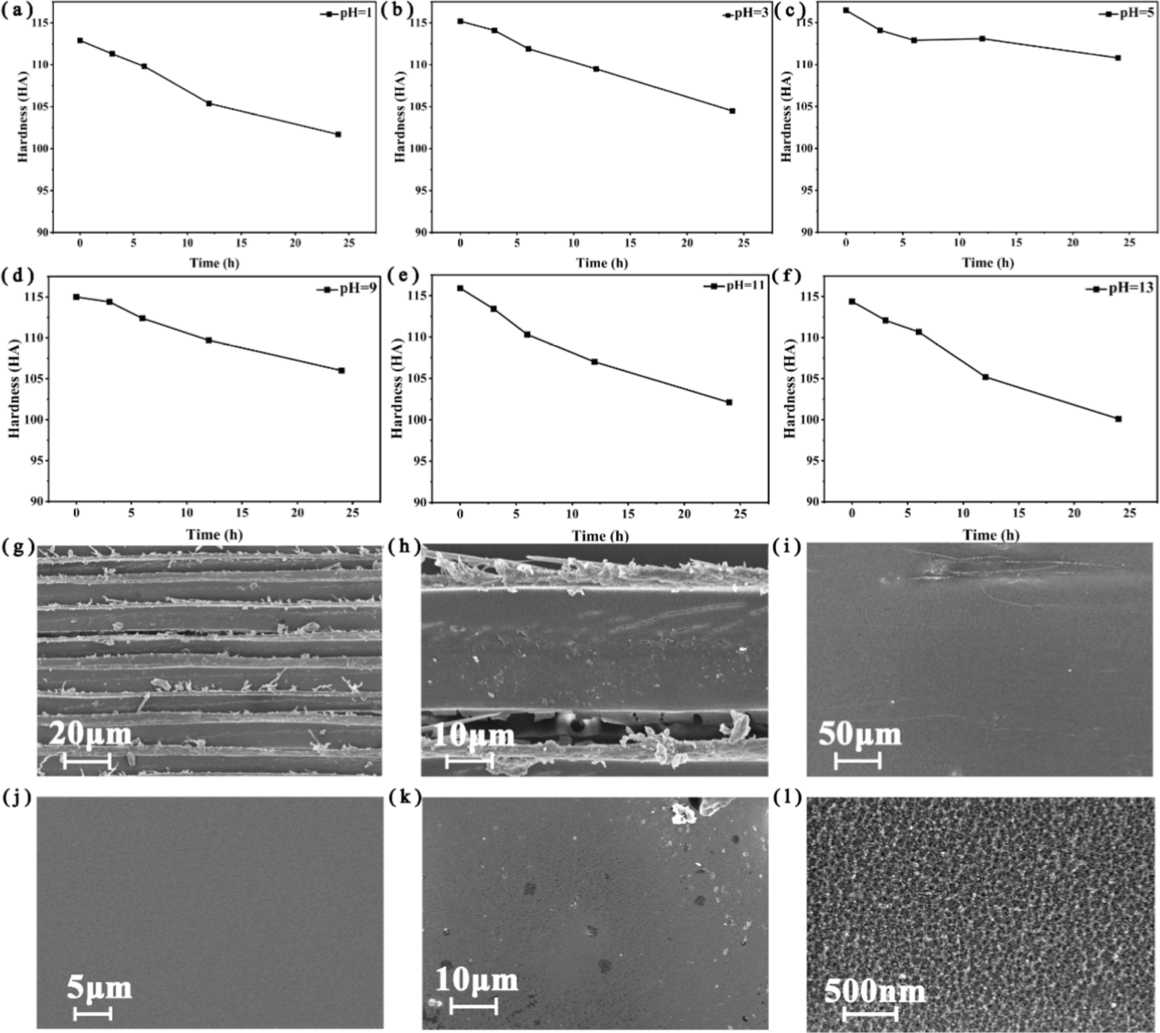
Changes in water contact angles of modified wood after immersion in solutions with different pH values (mass ratio of PMHS to DVB: 3:1; pH = (a) 1, (b) 3, (c) 5, (d) 9, (e) 11, and (f) 13). (g–h) Surface morphology of untreated wood. (i–j) Surface morphology of wood modified with the organosilicon/organic hybrid resin coating. (k–l) Surface morphology of wood modified with the organosilicon/organic hybrid resin coating after alkali corrosion.

Figure 4g–l presents the surface SEM images of untreated wood, modified wood with the hybrid coating, and modified wood after alkali corrosion, respectively. As shown in Figure 4g, the surface of untreated wood exhibits numerous rough groove-like structures, and at higher magnification, pits are observed between the grooves (Figure 4h), rendering the wood highly susceptible to moisture absorption from the environment and leading to deformation and decay. In contrast, the surface of wood modified with the organosilicon/organic hybrid resin coating is smooth and dense (Figure 4i–j), with the coating uniformly covering the wood surface and the original rough structures no longer observable, endowing the modified wood with excellent water resistance. As shown in Figure 4k, after alkali corrosion, uniformly distributed micropores appear on the surface of the modified wood, and a loose porous structure is observable at a higher magnification (Figure 4l), which corresponds to the organic resin framework that remains undissolved by the alkali. The SEM results demonstrate that the organosilicon/organic hybrid resin coating indeed exhibits an excellent alkali corrosion resistance.

### 3.5 Transparency and Color Regulation of the Hybrid Resin Coating

A UV–vis spectrophotometer was employed to characterize the transparency of the organosilicon/organic hybrid resin coatings, which are deposited onto a glass surface. Figure 5a shows the transmittance curves of hybrid resin coatings on glass with different PMHS/DVB mass ratios. As illustrated in Figure 5a, the text behind the glass slide coated with the hybrid coating was clearly visible (the pink material at the edge of the glass slide was tape used to prevent the coating solution from spreading), intuitively confirming that the hybrid coating possesses excellent transparency. The measurement results indicate that the transmittance of the hybrid coating is approximately 90% in the visible light region and exceeds 85% in the infrared region. When applied to the wood surface, this coating can protect the wood while preserving its natural texture and color, fully aligning with the core principle of “restoring the old as it was” in the field of wooden cultural relics and ancient architectural conservation.

**Figure 5:**
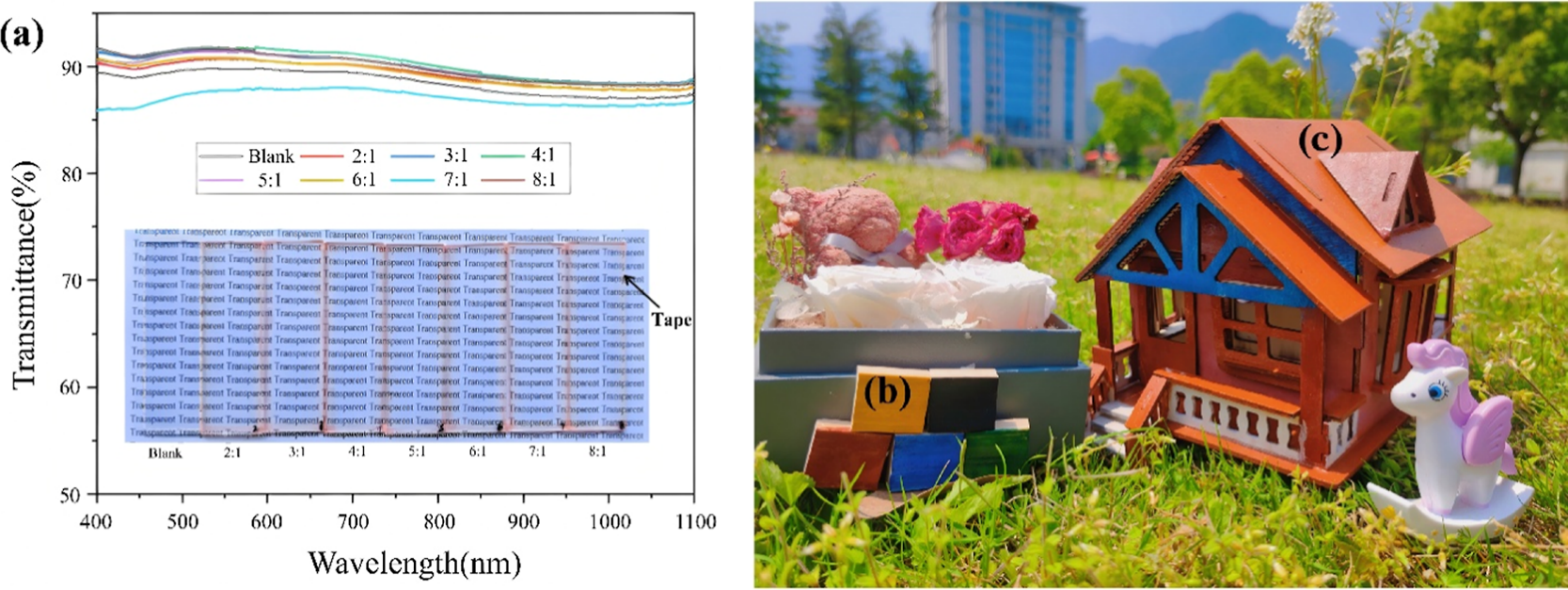
(a) Transmittance curves of hybrid resin coatings on glass with different PMHS/DVB mass ratios. (b) Wood blocks and (c) wooden house brushed with colored hybrid coatings.

Currently, the main colors of traditional timber-framed architecture in China are yellow, green, blue, and red. To this end, corresponding inorganic pigments were added to the organosilicon/organic hybrid resin solutions to prepare colored hybrid resin coatings, which were then brushed onto the surfaces of small wooden house models and wood block specimens to validate the application of colored coatings in areas of traditional wooden architecture, where color has worn away. As shown in Figure 5b,c, by selecting inorganic pigments of different colors, the organosilicon/organic hybrid resin coating can be matched with the commonly used color schemes of traditional ancient architecture, further enhancing the application potential of this coating in the field of ancient architecture and wooden cultural relic protection.

## 4 Conclusions

Based on the hydrosilylation click reaction between the Si–H bonds of PMHS and the vinyl groups of DVB, as well as the dehydrogenative click reaction between the Si–H bonds of PMHS and the –OH groups on the wood surface, an organosilicon/organic hybrid resin coating was constructed in situ on the wood surface. When the PMHS/DVB mass ratio was 3:1, the curing temperature was 100 °C, and the curing time was 72 h, the resulting coating exhibited excellent comprehensive performance. Compared with untreated wood, the water resistance of wood modified with the hybrid coating was significantly improved, with the water absorption rate decreasing from 78.6% to 13.89%. In comparison with silicone resin coatings (2940 cm), the total abrasion length of the organosilicon/organic hybrid resin coating reached 4300 cm, demonstrating superior abrasion resistance. Moreover, compared with traditional silicone coatings, the hybrid coating also exhibited excellent alkali resistance, as the modified wood maintains hydrophobicity even after immersion in an alkaline solution for 24 h. Additionally, the hybrid coating possessed favorable UV resistance, suggesting significant applications in outdoor environments.
